# Antibiotics versus appendectomy for uncomplicated acute appendicitis: a time-dependent framework for patient selection, treatment failure, and surgical rescue

**DOI:** 10.1097/MS9.0000000000005747

**Published:** 2026-10-06

**Authors:** Omar Mohamed Mohamed Kamaleldin Elsorougi, Shabbir Ahmad

**Affiliations:** a Emergency General Surgery Unit, Aintree Hospital, Liverpool University Hospitals NHS Foundation Trust, Liverpool, UK; b Department of General Surgery, Nangarhar University, Jalalabad, Afghanistan

**Keywords:** acute appendicitis, antibiotic therapy, appendectomy, appendicolith, nonoperative management

## Abstract

Antibiotic therapy has emerged as an evidence-supported alternative to appendectomy for selected adults with imaging-confirmed uncomplicated acute appendicitis. Recent individual patient data indicate that approximately two-thirds of antibiotic-treated patients avoid appendectomy during the first year. However, treatment is less definitive when an appendicolith is present, with higher rates of complications and subsequent appendectomy. Long-term evidence provides an important counterbalance, as the 10-year follow-up of the Appendicitis Acuta (APPAC) trial suggests that more than half of carefully selected patients may retain their appendix. These findings establish antibiotics as an effective but probabilistic strategy rather than an equivalent substitute for definitive source control. Importantly, appendectomy after initiating antibiotics is a heterogeneous outcome that may reflect early biological nonresponse, later recurrence, patient preference, or clinician thresholds for operative rescue. It should not automatically be interpreted as progression to complicated disease or clinically dangerous treatment failure. Appropriate selection must therefore incorporate diagnostic confidence, imaging features, physiologic stability, informed patient preferences, reliable follow-up, and timely access to surgery. This editorial proposes a time-dependent, risk-stratified framework that separates eligibility for nonoperative management from early treatment response, future recurrence risk, and the feasibility of prompt surgical rescue.

## Introduction

For more than a century, acute appendicitis was regarded as an inevitably progressive surgical disease, making appendectomy the default means of achieving definitive source control. Randomized evidence has since shown that many adults with imaging-confirmed uncomplicated appendicitis can be managed without immediate surgery^[^1–3^]^. Uncomplicated appendicitis is therefore increasingly understood not simply as an early stage on an unavoidable path to perforation, but as a heterogeneous inflammatory condition that may resolve with nonoperative treatment in appropriately selected patients^[^3,4^]^.

This shift has replaced therapeutic certainty with a more demanding clinical choice. Appendectomy provides near-definitive treatment and minimizes recurrence but exposes every patient to anesthesia, operative morbidity, and postoperative recovery. Antibiotics allow many patients to avoid or defer surgery, yet carry meaningful risks of primary nonresponse, subsequent appendectomy, and later recurrence. Approximately two-thirds of antibiotic-treated adults avoid appendectomy during the first year, while 10-year data from the Appendicitis Acuta (APPAC) trial suggest that more than half of carefully selected patients may retain their appendix over the longer term^[^1,2^]^. Neither result identifies the preferable strategy for an individual patient. This editorial therefore proposes a time-dependent, risk-stratified framework that separates eligibility for nonoperative management from the probability of early treatment failure and the feasibility of timely surgical rescue.

## What the evidence now establishes

Randomized evidence does not identify a universal treatment winner. In a 2025 individual patient data meta-analysis of six trials involving 2101 adults, 33.9% of patients assigned to antibiotics underwent appendectomy within 1 year, meaning that approximately two-thirds avoided surgery[1]. Overall complication rates were 5.4% with antibiotics and 8.3% with appendectomy, although the between-group estimate was imprecise. Outcomes differed substantially when an appendicolith was present. In this subgroup, 48.7% underwent appendectomy within 1 year, compared with 30.6% of patients without an appendicolith. Complications were also more frequent with antibiotics than with appendectomy among appendicolith-positive patients, at 15.0% versus 6.3%[1].

Long-term evidence provides a complementary perspective. At 10 years, the APPAC trial reported a histologically confirmed recurrence rate of 37.8% and a cumulative appendectomy rate of 44.3%, indicating that more than half of carefully selected antibiotic-treated patients retained their appendices[2]. These findings apply primarily to adults aged 18–60 years with computed tomography-confirmed uncomplicated disease and no appendicolith. In the more pragmatic Comparison of Outcomes of Antibiotic Drugs and Appendectomy (CODA) trial, antibiotics were noninferior to appendectomy for 30-day general health status, but 29% of antibiotic-treated participants underwent appendectomy within 90 days, including 41% with an appendicolith and 25% without one[5]. Antibiotics are therefore effective but probabilistic, whereas appendectomy is definitive but invasive.

## Who should be offered antibiotics?

Eligibility should begin with confidence in the disease phenotype rather than patient preference alone. Antibiotics are most defensible when imaging confirms uncomplicated appendicitis without an abscess, perforation, or suspected neoplasm; the clinical examination shows no diffuse peritonitis; and the patient is physiologically stable, able to tolerate treatment, and has reliable access to reassessment and urgent surgery[4]. Patients meeting these conditions, particularly those without an appendicolith who strongly value avoiding an operation, constitute a lower-risk group. Factors such as larger appendiceal diameter, greater inflammatory burden, older age, diagnostic uncertainty, major comorbidity, or constrained follow-up define an intermediate zone in which the choice should be individualized. Suspected gangrene or perforation, an abscess, sepsis, clinical deterioration, concern for malignancy, or inability to secure timely reassessment should generally shift treatment toward appendectomy.

Among candidate predictors, the appendicolith has the strongest and most consistent evidence. In a secondary analysis of CODA, it was associated with nearly twice the adjusted odds of appendectomy within 30 days after antibiotics (adjusted odds ratio, 1.99; 95% confidence interval, 1.28–3.10)[6]. Female sex and increasing appendiceal diameter were also associated with early appendectomy, whereas fever, leukocytosis, age, and comorbidity were not independent predictors. These exploratory associations should inform discussion rather than dictate treatment. No adequately validated bedside score currently estimates an individual patient’s probability of antibiotic failure. The proposed three-zone model should therefore be regarded as a clinical decision structure that integrates imaging, physiologic stability, patient priorities, and rescue capacity, not as a new prediction rule.

## Treatment failure is time-dependent

Treatment failure should be interpreted along a temporal continuum rather than as a single binary outcome. During the first 24–48 hours, persistent or worsening pain, evolving peritoneal signs, fever, hemodynamic disturbance, inability to tolerate oral therapy, or increasing inflammatory markers should prompt clinical reassessment. At this stage, the principal concerns are misclassified complicated appendicitis and failure to achieve source control. Because universally validated thresholds for conversion to surgery are lacking, decisions should integrate the patient’s clinical trajectory with repeat examination, laboratory findings, and imaging when appropriate[4].

The second phase comprises early step-up appendectomy after antibiotics have been initiated. Such surgery may represent biological nonresponse, but it may also reflect patient anxiety, clinician concern, or a deliberately low threshold for rescue. In the CODA analysis, 21% of antibiotic-treated participants underwent appendectomy within 30 days. Among those with an available reason, 77% underwent surgery for an acute clinical indication, whereas 21% did so for nonclinical reasons, including concern about recurrence or preference for surgery[6]. Appendectomy after antibiotics should therefore not automatically be equated with disease progression, perforation, or clinically dangerous treatment failure.

Once the index episode resolves, the clinical problem changes from acute nonresponse to recurrence risk. In the APPAC trial, the histologically confirmed recurrence rate was 32.4% at 5 years and 37.8% at 10 years, suggesting that recurrence accumulated predominantly during the earlier years and subsequently slowed[2]. Patients should therefore be counseled using absolute, time-specific estimates rather than the imprecise warning that appendicitis “may return.” Distinguishing immediate nonresponse, early step-up surgery, and late recurrence provides a more clinically meaningful account of what treatment failure actually means.

## Selection must include the healthcare environment

Favorable imaging alone does not make a patient suitable for nonoperative treatment. Antibiotics-first care requires physiologic stability, adequate pain control, the ability to tolerate oral medication and maintain hydration, clear return precautions, reliable communication and transport, and access to urgent reassessment and appendectomy. It also requires a patient who understands that avoiding surgery initially does not guarantee permanent avoidance of surgery. In this context, rescue capacity is not merely a logistical consideration; it is part of patient selection.

A secondary CODA analysis found outpatient antibiotic management to be feasible in selected adults. Serious adverse events during the first 7 days occurred at rates of 0.9 per 100 outpatients and 1.3 per 100 inpatients, while outpatients missed fewer workdays[7]. However, outpatient and inpatient management were not randomly assigned, and patients selected for discharge may have differed in clinically important ways. These findings should therefore support rather than replace careful assessment. A strategy that is reasonable for a stable patient living near an emergency surgical service may be inappropriate for an otherwise similar patient facing delayed transport, unreliable follow-up, or limited access to operative rescue. The safety of antibiotics-first treatment consequently depends not only on disease biology but also on the system in which uncertainty is being managed.

## Beyond antibiotics versus surgery

The conventional comparison between antibiotics and appendectomy may itself be incomplete. In the small Appendicitis Acuta III (APPAC III) trial, 10-day treatment success was 97% with antibiotics and 87% with placebo among 66 adults with computed tomography-confirmed uncomplicated appendicitis without an appendicolith, perforation, abscess, or suspected tumor. Antibiotics were not statistically superior to placebo, but the trial was underpowered, and failure to demonstrate superiority should not be interpreted as evidence of equivalence[3]. At 3 years, appendectomy and histologically confirmed recurrence rates also did not differ significantly between groups[8]. These findings support the possibility that some carefully defined cases represent a self-limiting inflammatory phenotype, but they do not justify routine supportive care alone.

The stewardship implication, therefore, is not that antibiotics should be abandoned, but that their necessity, spectrum, route, and duration require closer scrutiny. Future trials should determine which patients genuinely require antimicrobial therapy, whether shorter or narrower regimens provide comparable outcomes, and whether observation alone can be safely evaluated in a highly selected subgroup. The next phase of research may consequently extend beyond the antibiotics-versus-surgery comparison toward identifying the minimum effective intervention for different appendicitis phenotypes.

## Conclusion

Antibiotics and appendectomy should not be presented as interchangeable options for every patient with uncomplicated acute appendicitis. Antibiotics are most appropriate when uncomplicated disease is confidently established, the risk and consequences of nonresponse are acceptable, patient preferences are informed, and timely reassessment and operative rescue are available. Appendectomy remains preferable when diagnostic uncertainty, an appendicolith, clinical deterioration, or limited rescue capacity increases the risk of delayed source control. The central question is therefore not simply whether antibiotics can avoid surgery, but whether the uncertainty inherent in nonoperative treatment can be accepted, monitored, and resolved safely for the individual patient.

## Ethical approval

Not applicable.

## Consent

Not applicable.

## Data Availability

No new data were generated or analyzed for this article. All information discussed in the manuscript was obtained from the cited published literature.

## Research registration unique identifying number (UIN)

Not applicable. This article is an editorial and did not involve a prospectively registered research study.
